# PCSK9 Levels Are Raised in Chronic HCV Patients with Hepatocellular Carcinoma

**DOI:** 10.3390/jcm9103134

**Published:** 2020-09-28

**Authors:** Silvano Fasolato, Sabrina Pigozzo, Patrizia Pontisso, Paolo Angeli, Massimiliano Ruscica, Edoardo Savarino, Sara De Martin, Maria Giovanna Lupo, Nicola Ferri

**Affiliations:** 1 Department of Medicine, University of Padova, 35128 Padua, Italy; silvano.fasolato7@gmail.com (S.F.); sabrina.pigozzo@unipd.it (S.P.); patrizia.pontisso@unipd.it (P.P.); paolo.angeli@unipd.it (P.A.); 2 Department of Pharmacological and Biomolecular Sciences, University of Milan, 20122 Milan, Italy; massimiliano.ruscica@unimi.it; 3 Division of Gastroenterology, Department of Surgery, Oncology and Gastroenterology, University of Padua, 35128 Padua, Italy; edoardo.savarino@unipd.it; 4 Department of Pharmaceutical and Pharmacological Sciences, University of Padova, 35131 Padua, Italy; Sara.demartin@unipd.it (S.D.M.); mariagiovanna.lupo@phd.unipd.it (M.G.L.)

**Keywords:** PCSK9, hepatocellular carcinoma, HCV, cholesterol

## Abstract

*Background:* Since emerging evidence suggests a protective role of proprotein convertase subtilisin/kexin type 9 (PCSK9) on hepatitis C virus (HCV) infection, the aim of the present study was to evaluate the correlation between PCSK9 and HCV infection in hepatocellular carcinoma (HCC) patients. *Methods:* In this retrospective study, PCSK9 levels were evaluated by ELISA, in plasma samples from control (*n* = 24) and 178 patients diagnosed for HCC, cirrhosis, or chronic hepatitis, either positive or negative for HCV. *Results:* HCV positive patients (HCV+) presented with higher PCSK9 levels compared to HCV negative individuals (HCV-), 325.2 ± 117.7 ng/mL and 256.7 ± 139.5 ng/mL, respectively. This difference was maintained in the presence of HCC, although this disease significantly reduced PCSK9 levels. By univariate analysis, a positive correlation between PCSK9 and HCV viral titer was found, being G2 genotype the most-potent inducer of PCSK9 among other genotypes. This induction was not associated with changes in total cholesterol (TC), low-density lipoprotein cholesterol (LDL-C) and triglycerides (TG). A negative correlation was also found between PCSK9 levels and liver impairment, assessed by Model for End-Stage Liver Disease (MELD). Finally, a multivariate correlation analysis corrected for age, TC, LDL-C, and sex, demonstrated, in the whole cohort, a positive association between PCSK9 and HCV and a negative with HCC. *Conclusions:* taken together, our study reveals that HCV raised PCSK9 in both the presence and absence of HCC.

## 1. Introduction

Hepatocellular carcinoma (HCC) is a malignancy representing about 90% of primary liver cancers, and it is the third leading cause of cancer-related death worldwide [1]. Chronic liver diseases, either associated with hepatitis B (HBV) or hepatitis C (HCV) viruses, or alcoholic liver disease (ALD) or nonalcoholic fatty liver disease (NAFLD) are major risk factors for HCC [2]. Worldwide, HCV is the most common cause of HCC [3]. It has been found in 44%–66% of patients with HCC in Italy, and in 80% of HCC cases in Japan [4,5].

An intriguing interplay between HCV and the lipid metabolism of the host has been identified at molecular and cellular levels [6]. Apolipoproteins (apo) and neutral lipids affect the way HCV enters the host cell and generate a productive infection since changes in the intracellular membrane lipid composition by viral proteins is an indispensable step for assuring an adequate environment for viral replication. Furthermore, viral assembly is associated with lipid droplets and is dependent on the presence of apoE, and the viral particle seems to follow a similar maturation pathway as very-low-density lipoprotein (VLDL). In light of these considerations, it is not surprising that hepatic fat accumulation in obesity-related NAFLD is becoming a leading cause of HCC, especially in the USA and other Western countries [7].

Thus, the unique interaction between HCV and lipid metabolism offers the opportunity to investigate the role of key players in cholesterol homeostasis in HCV-associated HCC. Proprotein convertase subtilisin/kexin type 9 (PCSK9) represents the newest and more effective regulator of low-density lipoprotein cholesterol (LDL-C) levels. PCSK9 is mainly secreted by the liver, where it downregulates the LDL receptors (LDLRs) on the cell surface of hepatocytes by a post-translational mechanism [8]. This biological action determines an impairment of the LDL particles’ uptake by the liver, thus increasing levels of LDL-C.

Starting from this evidence, in the present study, we have analyzed a possible relationship between PCSK9 plasma levels and the positivity to HCV in HCC patients. The rationale of performing this analysis is supported by our previous study showing a positive association between plasma levels of PCSK9 with steatosis grade, necroinflammation, ballooning, and fibrosis stage in NAFLD patients [9]. Secondly, four hepatocyte surface proteins involved in HCV entry, such as the cluster of differentiation 81 (CD81), LDLR, VLDL receptor (VLDLR), and scavenger receptor class B type 1 (SR-B1), are negatively regulated by PCSK9 [10,11,12,13,14]. Finally, the mutual interaction between HCV and PCSK9 is supported by the fact that its promoter activity is induced in response to infection [15] and that PCSK9 inhibits HCV replication [16].

## 2. Material and Methods

### 2.1. Participants

178 Italian patients with established chronic liver disease were recruited prospectively and consecutively from the Department of Medicine of Padua University Hospital between 2011 and 2016. The inclusion criteria were as follows: (a) diagnosis of hepatocellular carcinoma, based on AASLD radiological criteria or histology, cirrhosis or chronic hepatitis either positive or negative for hepatitis C viruses.; (b) none of them underwent antiviral, chemotherapy, or surgical treatments; (c) no statin treatment; (d) non-active potus for at least 6 months; (e) no infections, with the exception of the viral infection by HCV; (f) no acute hepatitis or ongoing acute complications, such as encephalopathy and gastrointestinal bleeding; (g) no diabetes mellitus and dyslipidemia; (h) absence of autoimmune-diseases; (i) no treatments with antibiotics, immune-suppressive or anti-inflammatory drugs; (l) no cardiovascular disease (stroke, ischemic cardiopathy).

Serum was isolated from whole blood following clotting and centrifugation and stored immediately at −70 °C until thawed for analyses. For each patient, demographic and anthropometric features and serum lipids were determined. Plasma lipids (total and HDL cholesterol and triglycerides) were measured by certified enzymatic techniques. LDL cholesterol was calculated by the Friedewald’s equation. None of the 178 patients received statins as lipid-lowering therapy. Liver function was assessed by the Model for End-Stage Liver Disease (MELD). HCV RNA levels were measured by the COBAS^®^ AmpliPrep/COBAS^®^ TaqMan^®^ HCV Test, v.2.0 assay (Roche Molecular Systems, Pleasanton, CA, USA) with a lower limit of quantification of 25 IU/mL. HCV genotyping was assessed by RealTime HCV genotype II assay (Abbott Molecular Inc., Green Oaks, IL, USA). The study was approved by the Ethical Committee of the Padua University Hospital with Protocol Number: 1958P. Informed written consent was obtained from each patient, and the study conformed to the Ethical Guidelines of the 1975 Declaration of Helsinki.

### 2.2. PCSK9 Measurement

PCSK9 was blindly measured using a commercial ELISA kit (R & D Systems, MN, USA, cat. N° SPC900) with plasma aliquots collected after overnight fasting, stored at −80 °C, and diluted 1:20 as previously described [17]. As suggested, a 4-parameter logistic curve-fit was generated to obtain sample concentrations, using GraphPad Prism 5 (San Diego, CA, USA). The minimum detectable concentration was 0.219 ng/mL. Intra- and inter-assay CVs were 5.4% ± 1.2% and 4.8% ± 1.9%, respectively.

### 2.3. Statistical Analysis

Spearman’s rank correlation was used to measure statistical dependence between 2 variables. A comparison between data groups was performed using the non-parametric Mann Whitney U test, and, when the comparison was carried out in more than 2 groups, the Kruskal-Wallis analysis of variance was performed. The analysis was performed using GraphPad InStat 3.0 software (San Diego, CA, USA). Multivariate analysis was carried out using PCSK9 as a dependent variable and age, sex, total cholesterol, LDL-C, HCV, MELD, and HCC as an independent variable. The calculations were performed using IBM SPSS statistics 26 (Armonk, NY, USA). Significance was set as *p* < 0.05.

## 3. Results

In the present observational study, we measured the plasma levels of PCSK9 from control (*n* = 24), HCC patients HCV positive (*n* = 53) or negative (*n* = 33), and not HCC patients, positive (*n* = 73) or negative (*n* = 19) for HCV for a total of 178 patients. The anthropometric characteristics are shown in Table 1.

Considering the PCSK9 levels in each subgroup, we observed a significant increase in its levels in chronic hepatitis HCV positive (HCV+) compared to controls (Table 1). In addition, the control group had higher total cholesterol (TC), LDL-C, and triglycerides (TG) (Table 1).

**Figure 1:**
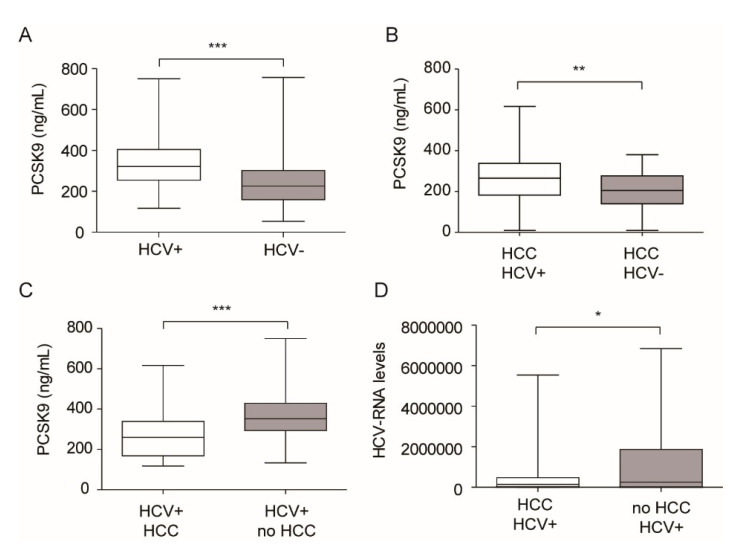
HCV infection is associated with higher levels of circulating PCSK9, irrespectively from the presence of HCC. (**A**) Whole HCV+ population showed increased PCSK9 plasma levels compared to HCV- patients; (**B**) Plasma PCSK9 was found higher in HCC HCV+ subgroup compared to HCC HCV- subgroup; (**C**) HCC HCV+ patients showed a decrease in PCSK9 plasma levels compared to no HCC HCV+ patients; (**D**) HCC HCV- subpopulation showed a higher viral titer compared to HCC HCV+ group. * *p* < 0.05; ** *p* < 0.01; *** *p* < 0.001.

When we divided our patient population exclusively on HCV infection, we found that HCV+ showed significantly higher plasma levels of PCSK9 compared to HCV negative (HCV-) individuals, 325.2 ± 117.7 vs. 256.7 ± 139.5 ng/mL (*p* < 0.001) (Figure 1A). Similar results were found in the HCC subgroup, 276.1 ± 127.9 and 206.9 ± 89.9 (Figure 1B). On the contrary, when considering the HCV+ patients exclusively, we observed that the presence of HCC significantly reduced PCSK9 levels (277.1 ± 126.1 vs. 357.6 ± 110.1; *p* < 0.001) (Figure 1C). Thus, the positivity to HCV induced PCSK9 in the circulation, independently from the presence of HCC.

We also observed a higher viral titer in HCV+ patients not affected by HCC, compared to HCC patients (Figure 1D). Thus, the differences observed between these two groups of patients may be derived from the HCV-dependent induction of PCSK9 or by the negative effect of HCC on liver functionality and thus on PCSK9 neo-synthesis and secretion into the circulation.

**Figure 2:**
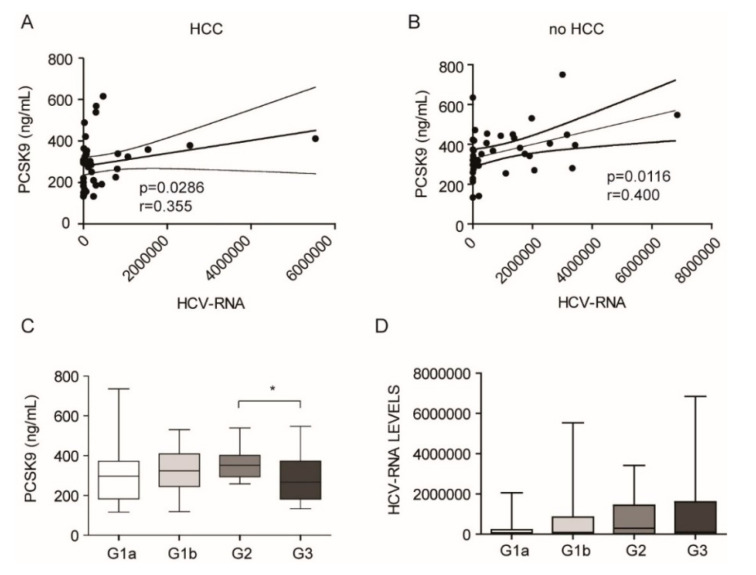
Association between PCSK9 plasma levels and HCV viral titer and genotype. (**A**,**B**) linear regression analysis, with a 95% confidence interval, between PCSK9 levels and HCV viral titer in HCC (**A**) and no HCC patients (**B**). (**C**) PCSK9 levels in different HCV genotypes. (**D**) Viral titer levels between HCV genotypes. * *p* < 0.05.

To address this issue, we analyzed the possible correlation between the HCV viral titer and PCSK9 levels in both HCC and no HCC patients. As shown in Figure 2, PCSK9 levels positively correlated with viral titer in both subgroups of patients. These data suggest that HCV infection induced the synthesis of PCSK9 [15,18] independently from the presence of a hepatic tumor.

Additionally, the analysis of different HCV genotype demonstrated a non-significant trend of increased of PCSK9 levels from G1a, to G1b and G2 and a significantly lower plasma levels in G3 compared to G2 (Figure 2C). These data suggest that the G2 genotype was the most-potent inducer of PCSK9 expression, an effect that was independent of the viral titer levels, which did not differ between the four subgroups (Figure 2D).

**Figure 3:**
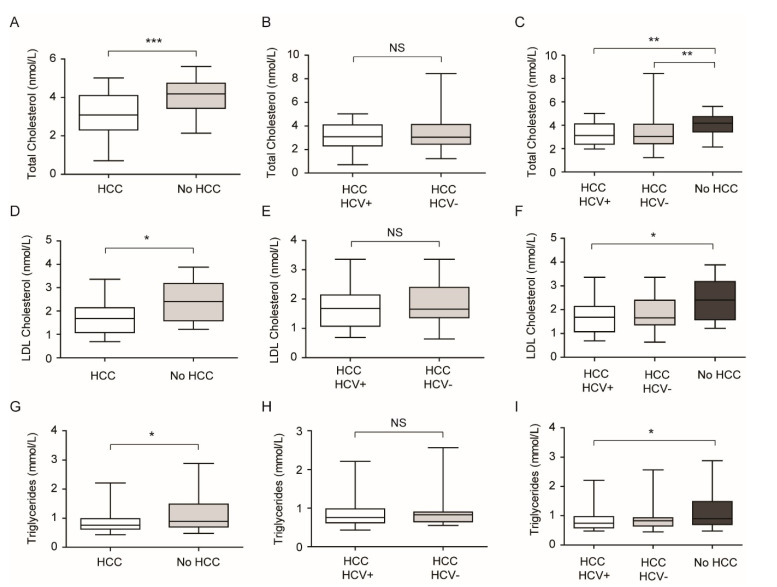
Cholesterol and TG levels in different patient subgroups. (**A,D,G**) The presence of HCC caused a decrease in TC (**A**), LDL-C (**D**) and TG (**G**) levels compared to no HCC patients; (**B,E,H**) no significant changes were observed between HCC HCV+ and HCC HCV- patients in TC (**B**), LDL-C (**E**) and TG (**H**) levels; (**C,F,I**) both HCC HCV+ and HCC HCV- subgroups showed lower TC (**C**), LDL-C (**F**) and TG (**I**) levels compared to no HCC population. * *p* < 0.05; ** *p* < 0.01; *** *p* < 0.001. NS: not significant.

Due to the relevant role of PCSK9 on lipid and cholesterol homeostasis and the interplay between lipids and HCV infection, we measured lipid parameters in our cohort. HCC patients showed a significant lower TC, LDL-C, and TG levels compared to non-HCC patients (Figure 3A,D,G). In HCC patients, the presence of HCV did not significantly alter both TC, LDL-C, and TG (Figure 3B,E,H), and both HCC groups (HCV- and HCV+) had lower lipid levels than patients without HCC (Figure 3C,F,I). These data indicated that the increased levels of PCSK9 in response to HCV infection was not sufficient to significantly affect the lipoprotein receptors (i.e., LDLR and VLDLR) and thus LDL-C and TG plasma concentrations. On the contrary, the hypolipidemic status observed in HCC patients compared to non-HCC patients might be due to significant liver impairment and thus apoB synthesis and VLDL production.

**Figure 4:**
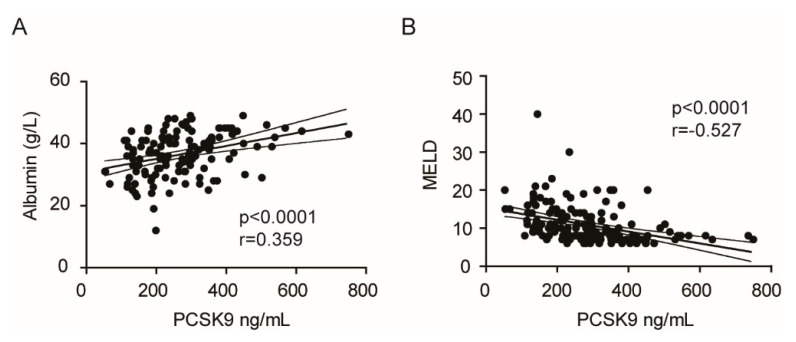
Linear regression analysis PCSK9 and liver functionality. (**A**,**B**) Linear regression analysis, with a 95% confidence interval, between PCSK9 levels and albumin (**A**) and MELD (**B**).

To explore this possibility, we performed a series of linear regression analyses. In all the population, a significant positive correlation between albumin levels and PCSK9 was observed (Figure 4A). In addition, we found a negative correlation between MELD, a score for liver disease, and PCSK9 (Figure 4B).

A correlation analysis evidenced that in HCC patients positive for HCV, PCSK9 plasma levels were positively correlated with albumin and viral titer, and negatively with MELD (Table S1). In contrast, the correlation between PCSK9 and liver function was lost in HCC patients negative for HCV (Table S2). These results indicated that HCV infection sustained the PCSK9 synthesis and secretion also in patients with severe liver impairment and protein synthesis, including albumin and PCSK9.

A more restricted multivariate correlation analysis that considered as independent variables age, gender, total cholesterol, LDL-C, HCV, and HCC, demonstrated in the all population a positive association between PCSK9 and HCV and a negative with HCC (Table 2).

## 4. Discussions

Since the discovery of the strong downregulatory action of PCSK9 on the LDLR [8] and CD81 [12], a protective role of PCSK9 on HCV infection of hepatocytes has been postulated [12] and thus, the treatment with monoclonal antibodies anti PCSK9 might increase the vulnerability to the infection [13].

In this regard, the HMG-CoA reductase inhibitors, statins, might also have pro-viral effects by increasing LDLR and Niemann-Pick C1-Like 1 (NPC1L1) expression. However, this hypothesis has not been confirmed in two cohort studies, including HCV patients showing a reduced risk of cirrhosis development, and incidence of HCC, for patients with statin use [19,20]. These apparent discrepant results could be related to the fact that statins may interfere with HCV replication by inhibiting the synthesis of isoprenoids of the mevalonate pathway, such as geranylgeraniol, which is necessary for viral replication [21]. In addition, the association of statin therapy with a net antiviral effect has been attributed to the downregulation of claudin-1 expression [22], as well as the induction of PCSK9 levels [23], which is associated with lower surface receptor expression [24].

Within this contest, in the present study, we have measured the PCSK9 plasma levels in patients infected by HCV in the presence or absence of HCC. The main finding was that plasma PCSK9 concentrations were significantly increased in the HCV-infected patients and that this induction was associated with increased viral titer levels. This increase was observed independently from the presence of HCC, a pathological condition that reduces protein synthesis, including albumin and PCSK9 [25]. Our results are in line with previous studies conducted in HCV infected patients [26]. More in detail, Hyrina et al. observed an increase of PCSK9 levels in patients who achieved sustained virologic response (SVR), suggesting that circulating PCSK9 may impede viral infection [26].

In addition, we found a positive correlation between the viral titer and PCSK9 levels, thus suggesting that HCV directly induces the viral replication. In vitro study supports this hypothesis, demonstrating that PCSK9 promoter activity is upregulated in response to HCV infection in cultured cells [15]. PCSK9 gene expression is regulated by the transcription factors SREBP1 and 2 and HNF1α [27,28,29]. The authors investigated their relative contribution to HCV-mediated PCSK9 promoter activity. The analysis demonstrated that transcription factors SREBP1c and HNF1α increased PCSK9 promoter activity in HCV replicon cells, whereas SREBP1a, HNF1β, and FoxO3 had an inhibitory effect [15].

Beyond the effect of HCV on PCSK9 plasma levels, a second point to take into consideration is the limiting effect of PCSK9 on HCV entry and replication [12,16]. Our retrospective analysis could not determine if patients with higher levels of PCSK9 are protected by HCV infection, thus in the future, it could be interesting to investigate this hypothesis in a prospective clinical study including also patients under treatment with PCSK9 inhibitors. In this regard, it is important to mention that alirocumab, an anti-PCSK9 monoclonal antibody, has been shown to not alter the CD81 receptor expression or HCV entry and replication into hepatocytes [13].

In the present study, we have also investigated the potential effect of the induction of PCSK9 by HCV on lipid profile. The results demonstrated that the positivity to HCV was not associated with significant changes in TC, LDL-C, and TG levels. Thus, it is possible that the extent of the induction of PCSK9 was not sufficient to downregulate the LDLR significantly. Alternatively, it is possible to envision a counterbalancing effect of HCV on LDLR. Indeed, HCV has been shown to stimulate the LDLR expression in both HCV-infected hepatic cells (Huh7) and liver tissue from chronic hepatitis C patients [18]. On the contrary, the presence of HCC significantly reduced TC, LDL-C, TG, and PCSK9. This effect is potentially due to a significant impairment of liver function that leads to lower protein synthesis, including apoB and PCSK9. Indeed, PCSK9 positively correlated with albumin and negatively with MELD.

Our analysis also pointed out a viral genotype-dependent effect on PCSK9 plasma levels. G2 infection appeared to be more effective in inducing PCSK9, and significantly lower levels were observed in the G3 phenotype. This result was shown to be independent of MELD that did not differ among different viral subgroups (Table S3. Thus, G3 infection might be associated with a hypolipidemic status in response to a lower amount of circulating PCSK9, a phenotype that has been previously described [30]. However, our analysis of plasma lipid and cholesterol concentration in HCV patients infected with different genotype did not find any statistically significant difference in terms of TC, LDL-C, and TG in G3 patients (Figure S1). In contrast, we found a significantly higher concentration of TC, TG, and LDL-C in G1b compared to G1a phenotype, although this difference may be guided by the higher percentage of patients with HCC in G1a infected patients (57% vs. 39% of HCC positive patients in G1a vs. G1b genotype) (Table S4).

## 5. Conclusions

In conclusion, our study reveals a complex interplay among HCC, HCV, and PCSK9 plasma levels confirming the potent inducing activity of HCV on PCSK9, particularly the G2 phenotype, even in patients with significant liver impairment, such as those with HCC. The protective effect of PCSK9 on viral entry still needs to be addressed.

## Figures and Tables

**Table 1:** Anthropometric and biochemical parameters of recruited patients. HCC: Hepatocellular carcinoma; HCV: Hepatitis C virus; BMI: Body mass index; TC: Total cholesterol; LDL-C: Low-density lipoprotein cholesterol; TG: Triglycerides. SEM: Standard error of the mean. ND: Not determined. ^a^ *p* < 0.05 vs. control; ^b^ *p* < 0.01 vs. control; ^c^ *p* < 0.001 vs. control; ^d^ *p* < 0.05 vs. HCC HCV+; ^e^ *p* < 0.01 vs. HCC HCV+; ^f^ *p* < 0.001 vs. HCC HCV+; ^g^ *p* < 0.01 vs. HCC HCV-; ^h^ *p* < 0.001 vs. cirrhotic HCV+; ^i^ *p* < 0.01 vs. cirrhotic HCV-; ^l^ *p* < 0.001 vs. cirrhotic HCV-; ^m^ *p* < 0.05 vs. HCC HCV-.

Parameters		HCC Patients (*n* = 86)		Not HCC Patients (*n* = 92)			
				Cirrhotic		Chronic Hepatitis	
	Control	HCV+	HCV-	HCV+	HCV-	HCV+	HCV-
N (M/F)	24 (9/15)	53 (39/14)	33 (28/5)	19 (15/4)	15 (11/4)	54 (29/25)	4 (2/2)
Age (years) Mean ± SEM	51.4 ± 9.6	64.6 ± 11.3 ^b^	66.6 ± 8.4 ^b^	62.8 ± 11.4	64.6 ± 7.3	57.1 ± 21.2 ^m^	66.1 ± 9.6
PCSK9 (ng/mL) Mean ± SEM	244.9 ± 74.2	271.76 ± 123	252.06 ± 130.06	307.9 ± 143.1	205.6 ± 105	357.9 ± 114.7 ^b,e,g,l^	330.6 ± 78
TC (mmol/L) Mean ± SEM	5.07 ± 0.9	3.21 ± 1.15 ^c^	3.39 ± 1.4 ^c^	2.8 ± 0.8 ^c^	2.95 ± 0.88 ^c^	4.1 ± 0.89 ^b,f,m,h,i^	3.58 ± 0.67
LDL-C (mmol/L) Mean ± SEM	2.7 ± 0.7	1.71 ± 0.82l ^c^	1.90 ± 0.7 ^b^	1.39 ± 0.8 ^c^	1.92 ± 0.77 ^a^	2.4 ± 0.85 ^f,h^	1.98 ± 0.88
TG (mmol/L) Mean ± SEM	1.47 ± 0.36	0.86 ± 0.36 ^c^	0.92 ± 0.40 ^c^	0.88 ± 0.20 ^c^	0.85 ± 0.31 ^c^	1.15 ± 0.64 ^d^	1.01 ± 0.32
Albumin (g/L) Mean ± SEM	ND	34.7 ± 7.3	36.5 ± 6.8	37.9 ± 6.0	34.7 ± 5.8	41.9 ± 5.6	37.5 ± 9.2

**Table 2:** Multivariate analysis of whole population.

Coefficients ^a^					
Model	UnstandardizedCoefficients		Standardized Coefficients	t	*p*-Value
	B	Std. Error	Beta		
1 (Constant)	261.811	84.762		3.089	0.003
Age	−0.859	0.852	−0.103	−1.009	0.316
Sex	−28.789	33.901	−0.091	−0.849	0.399
Total cholesterol	23.586	30.146	0.184	0.782	0.437
LDL-C	25.849	38.699	0.155	0.668	0.506
**HCV**	61.433	30.207	**0.210**	2.034	**0.046**
**HCC**	−80.080	30.388	**−0.281**	−2.635	**0.010**

^a^ Dependent Variable: PCSK9.

## Supplementary Materials

The following are available online at https://www.mdpi.com/2077-0383/9/10/3134/s1, Figure S1: Cholesterol and TG levels in patients HCV positive divided by virus genotype. (A) Total cholesterol; (B) TG; (C) LDL-C. Table S1: Correlation analysis in HCC HCV+ patients. Table S2: Correlation analysis in HCC HCV- patients. Table S3: MELD values between patients with different HCV genotype. Table S4: Percentage of HCC positivity in different genotype of HCV infected patients.
