# Supplementary material for: PCSK9 Levels Are Raised in Chronic HCV Patients with Hepatocellular Carcinoma
Source: J Clin Med. 2020 Sep 28;9(10):3134. doi: 10.3390/jcm9103134 (PMC7600304; doi:10.3390/jcm9103134)
Supplement: Supplementary file 1 [file jcm-09-03134-s001.pdf]

Journal of Clinical Medicine

MDPI

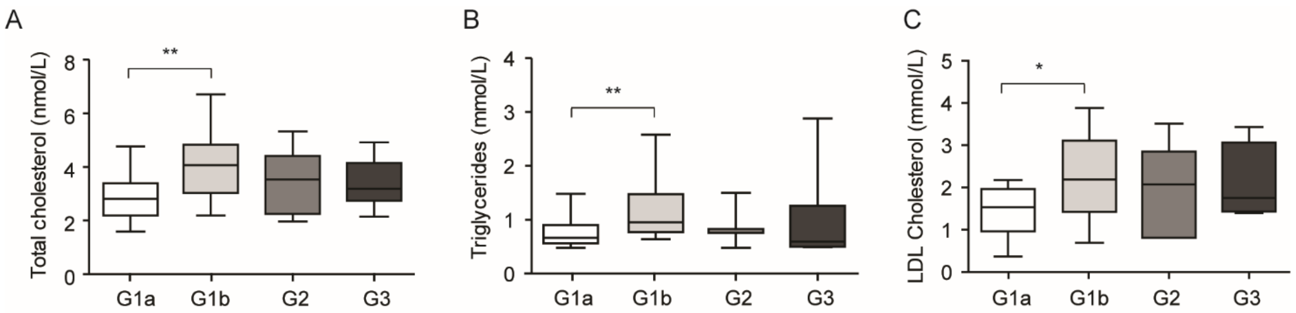

Figure S1. Cholesterol and TG levels in patients HCV positive divided by virus genotype. A) Total cholesterol; B) TG; C) LDL-C.

Table S1. Correlation analysis in HCC HCV+ patients.

r	Age	Albumin (g/dL)	MELD	Viral titer (UI/mL)	PCSK9 (ng/mL)
**Age**		−0.241	0.060	0.241	0.0004
**Albumin (g/dL)**	-0.241		**−0.544**	−0.055	**0.501**
**MELD**	0.060	−0.544		−0.247	**−0.585**
**Viral titer (UI/mL)**	0.241	−0.055	-0.247		**0.302**
**PCSK9 (ng/mL)**	0.0004	**0.501**	**−0.585**	**0.302**	

p-value	Age	Albumin (g/dL)	MELD	Viral titer (UI/mL)	PCSK9 (ng/mL)
**Age**		0.094	0.675	0.105	0.997
**Albumin (g/dL)**	0.094		**5.25 × 10⁻⁵**	0.721	**0.0003**
**MELD**	0.676	**5.25 × 10⁻⁵**		0.097	**7.8 × 10⁻⁶**
**Viral titer (UI/mL)**	0.105	0.721	0.096		**0.043**
**PCSK9 (ng/mL)**	0.997	**0.0003**	**7.81 × 10⁻⁶**	0.043	

Table S2. Correlation analysis in HCC HCV- patients.

r	Age	Albumin (g/dL)	MELD	PCSK9 (ng/mL)
**Age**		0.087	−0.074	−0.207
**Albumin (g/dL)**	0.087		**−0.530**	−0.008
**MELD**	−0.074	**−0.530**		0.135
**PCSK9 (ng/mL)**	−0.207	−0.008	0.135	

p-value	Age	Albumin (g/dL)	MELD	PCSK9 (ng/mL)
**Age**		0.641	0.692	0.255
**Albumin (g/dL)**	0.641		**0.002**	0.965

**MELD**	0.692	**0.002**		0.468
**PCSK9 (ng/mL)**	0.254	0.965	0.468	

Table S3. MELD values between patients with different HCV genotype.

Genotype	N	MELD (mean ± SD)
**2**	13	7.7±1.3
**3**	18	10.3±5.7
**1a**	16	10.6±4.0
**1b**	41	10.17±4.1

Table S4. Percentage of HCC positivity in different genotype of HCV infected patients.

	HCC (%)	NO HCC (%)
**G1a**	57	43
**G1b**	39	61
**G2**	15	85
**G3**	21	79
